# Does Obesity Make Transforaminal Lumbar Interbody Fusion More Difficult: A Retrospective Analysis

**DOI:** 10.7759/cureus.3762

**Published:** 2018-12-21

**Authors:** H. Bahadir Gokcen, Cagatay Ozturk

**Affiliations:** 1 Orthopedics, Istinye University, Istanbul, TUR

**Keywords:** obesity, body mass index, interbody fusion

## Abstract

Objective

To evaluate and compare peri-operative technical difficulties associated with single-level transforaminal lumbar interbody fusion (TLIF) and peri-operative outcomes between obese and non-obese patients.

Subjects and methods

The data, including age, blood transfusion volume, preoperative hemoglobin/hematocrit levels, operative time, blood loss, fluoroscopy time, skin incision length, and body mass index (BMI), of 53 patients undergoing single-level TLIF (L4–5 or L5–S1) between 2016 and 2018 were analyzed retrospectively. The patients were divided into two groups: BMI < 30 kg/m^2^ and BMI 30–39.9 kg/m^2^. Parameters were subjected to statistical analysis according to the BMI.

Results

There were 26 patients in the BMI < 30 kg/m^2^ group and 27 patients in the BMI 30–39.9 kg/m^2^ group. The average age of the patients was 60.8 years (30–70 years), and the average BMI was 29.9 kg/m^2^ (23.1–39.9 kg/m^2^). The fluoroscopy times and skin incision lengths were significantly different between the two groups (p < 0.05).

Conclusions

An experienced surgical team can safely apply the TLIF procedure in patients with obesity but it should be taken into consideration by surgeons before surgery. Some modifications in the surgical technique, including, further lateral dissection and wider skin incision in the TLIF technique for obese patients may be required during the procedure. This approach makes TLIF technique easier and safer in obese patients. The longer fluoroscopy times in obese patients indicate that more radiation exposure occurs during TLIF and that necessary precautions should be taken for maintaining surgical team and patient health.

## Introduction

Obesity is a health problem of increasing importance in developed and developing countries. Obesity, defined as a body mass index (BMI) >30 kg/m^2^, is associated with significant negative medical and financial outcomes, including lower back pain and higher risks of injury. Obesity is also associated with adverse effects on long-term surgical outcomes [1,2]. Obesity can cause spinal disease comorbidities and may increase the risk of postoperative complications. The clinically important BMI threshold and the effects of obesity in patients are still under investigation. With the increasing prevalence of obesity, the rate of surgical failures may increase [2]. Advances in technology have facilitated progress in spinal fusion techniques. The transforaminal lumbar interbody fusion (TLIF) technique is a generally accepted fusion operation for spinal surgery [3] that was first described by Harms and Rolinger in 1982 as a modification of the posterior lumbar interbody fusion technique [4]. This technique has several advantages over previous methods, including better clinical outcomes and theoretical advantages of larger healing surfaces and decreased need for spinal cord retraction during surgery [5,6]. However, the steep learning curve for TLIF poses a challenge to the surgical team when treating obese patients (BMI > 30 kg/m^2^). Excessive amounts of muscle and fatty tissue at the incision site have led to the design of longer specialized retractors to effectively retract these tissues during the TLIF procedure [7]. The main purpose of the present study was to evaluate the peri-operative difficulties and outcomes associated with single-level TLIF in obese patients.

## Materials and methods

**Figure 1:**
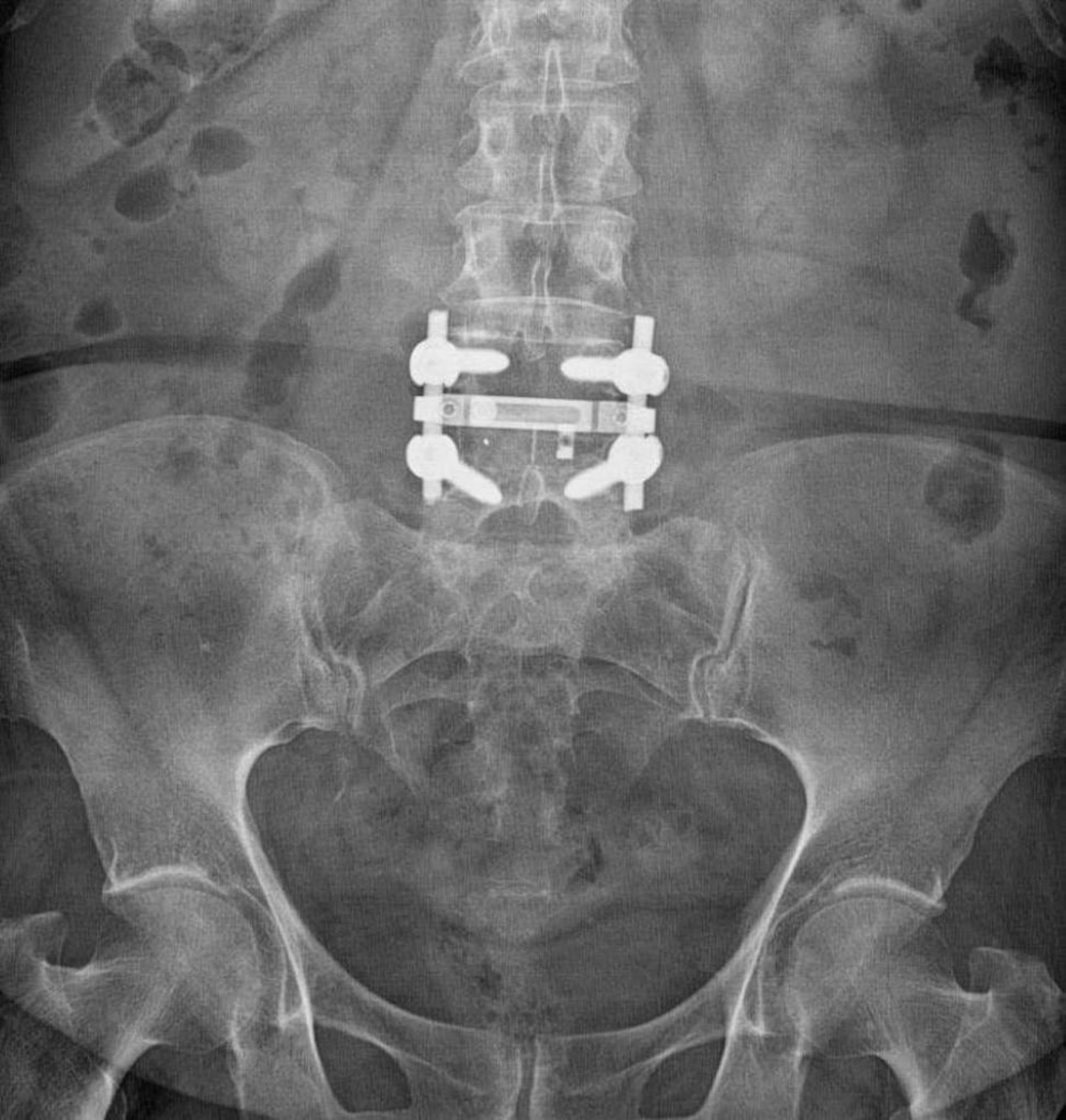
Postoperative radiograph of a patient with bilateral radiculopathy after a transforaminal interbody fusion procedure.

**Figure 2:**
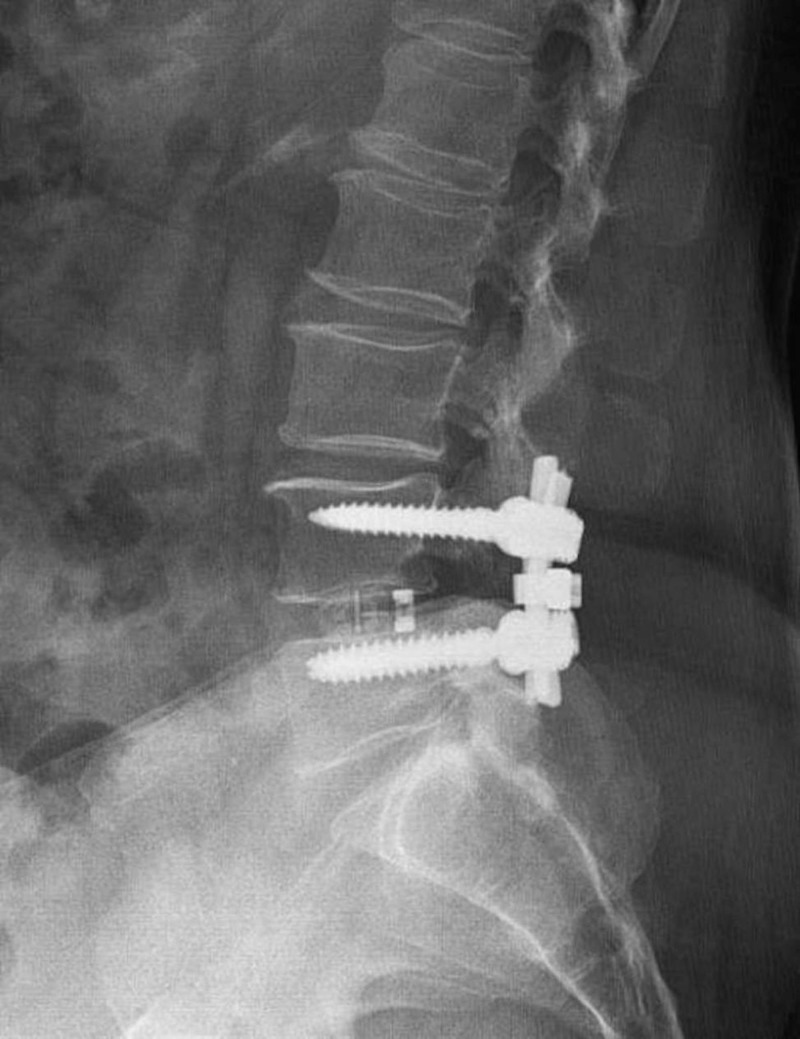
Postoperative radiograph of a patient with bilateral radiculopathy after a transforaminal interbody fusion procedure.

We analyzed the effects of BMI on the length of skin incision, blood loss, duration of surgery, volume of transfused blood, and duration of fluoroscopy in patients undergoing TLIF. Sixty-five patients over the age of 18 years who underwent single-level TLIF surgery between 2016 and 2018 by an experienced spinal surgeon were enrolled in this study. Ten patients were excluded because of comorbidities (malignancy and infection), and two were excluded because they had BMI > 39.9 kg/m^2^. The records of the remaining 53 patients were retrospectively analyzed. The preoperative radiological evaluation included anterior-posterior, lateral, and flexion-extension radiographs; lumbar computed tomography (CT) and magnetic resonance imaging (MRI). Standard general anesthesia was administered by the same anesthesia team. Four pedicle screws, two screws superior and two screws inferior to the segment of the pathological disc, were placed during the TLIF performed on the symptomatic side (Figures 1, 2).

The patient’s age, volume of blood transfusion, blood loss, preoperative hemoglobin (Hb) and hematocrit (Hct) levels, operative time, fluoroscopy time, skin incision length, and preoperative BMI (calculated by dividing the weight in kilograms by the square of the height in meters) were analyzed. Patients with BMI 30–39.9 kg/m^2^ were defined as obese according to the World Health Organization (WHO) classification [8]. The patients were divided into two groups: BMI < 30 kg/m^2^ and BMI 30–39.9 kg/m^2^. Parameters were subjected to statistical analysis according to the BMI.

Surgical technique

**Figure 3:**
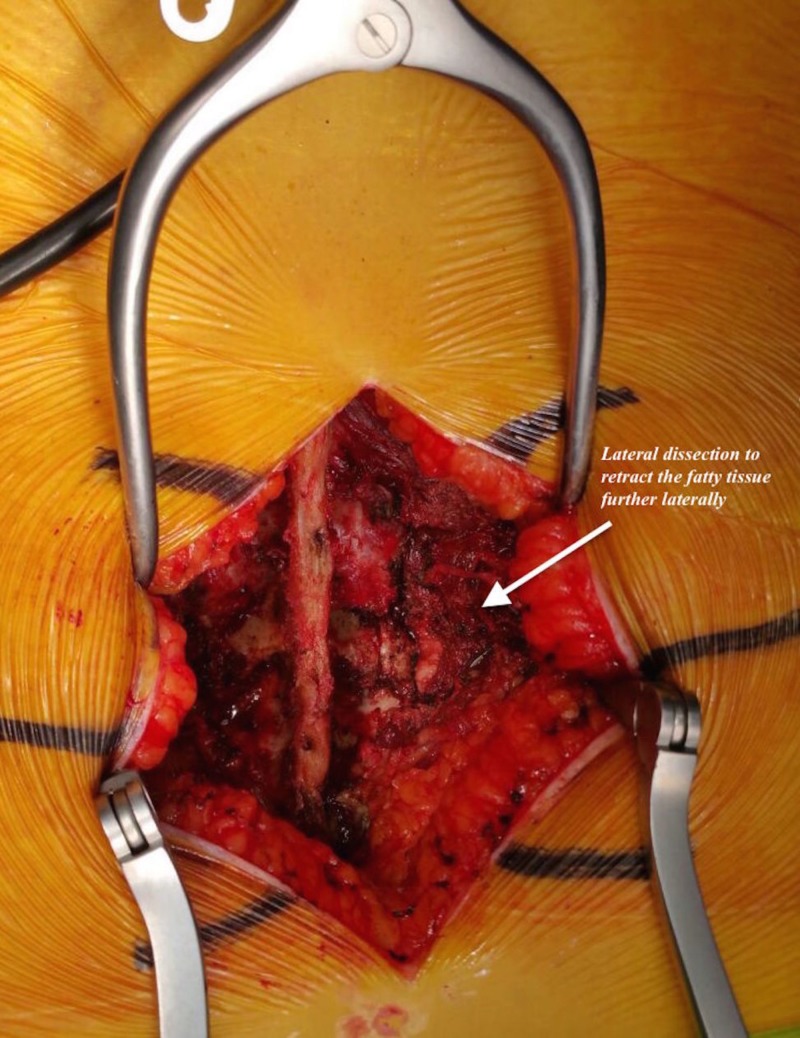
Lateral dissection in a patient in the body mass index 30-39.9 kg/m2 group.

**Figure 4:**
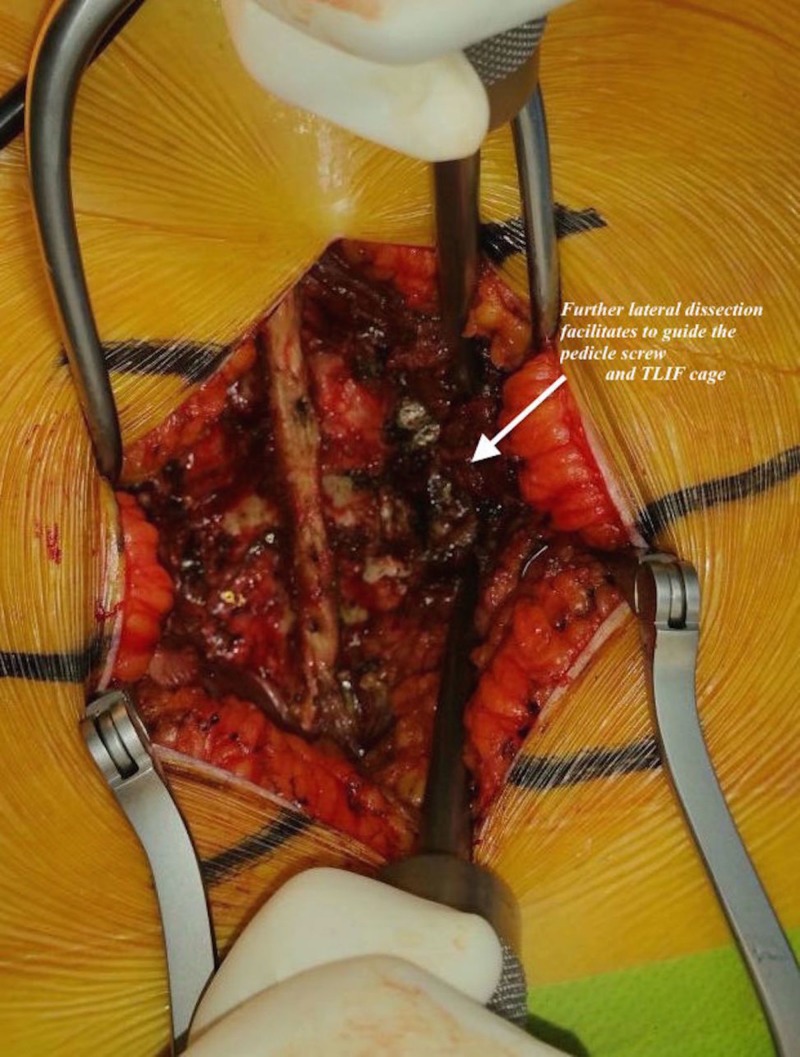
Lateral dissection in a patient in the body mass index 30-39.9 kg/m2 group.

Neuromonitoring was utilized in all cases. Each patient was placed on a radiolucent frame in the prone position and then supported with pillows to reduce the intraabdominal pressure. The patient’s head was fixed in a neutral position using silicon pillows. The axillary region and elbows were supported with pillows. The levels requiring surgery were identified using C-arm fluoroscopy and were marked with a marker pen. A posterior longitudinal skin incision was made after sterile surgical scrubbing was performed and proper surgical dressing was placed. The subcutaneous tissue was sharply dissected to reach the spinous processes. Starting from the spinous process, the lamina was located while taking care to protect the supraspinal and interspinal ligaments. In obese patients, we performed a lateral dissection to retract the subcutaneous fatty tissue more laterally in order to facilitate pedicle screw placement and TLIF cage placement (Figures 3, 4).

We made sure to protect the capsule of the facet proximal to the level of the disc undergoing TLIF. The Roy–Camille lumbar spine pedicle screw placement technique was followed when marking the pedicle screw entry point. A pin was used to mark the set point, and the entry point was verified using anteroposterior and lateral fluoroscopic imaging. The cancellous tissue of the pedicle was reached using a rongeur, and the pedicle was passed via a pedicle finder. Pedicle screws of appropriate size were placed following control of the pedicle walls via a probe. An appropriately beveled rod was placed opposite the area undergoing TLIF, which was adapted to the pedicle screws. Hemilaminectomy was performed on the side of the radiculopathy. In patients with bilateral radiculopathy, a total laminectomy was performed with bilateral foraminotomy. To gain access to the disc area, a partial inferior facetectomy was performed on the proximal facet joint, and a superior facetectomy was performed on the distal facet joint of the area undergoing unilateral fusion. The ligamentum flavum was cleaned using Kerioson. The disc space was reached, with care taken to protect the ascending and descending nerve roots. The epidural veins were coagulated using bipolar cautery. The disc was partially evacuated after opening the disc space with a size 15 scalpel. The disc space was expanded by distraction through the pedicle screws, and the remaining disc material was completely evacuated by reaching the contralateral disc from the TLIF side. The endplates of the superior and inferior vertebrae on both the TLIF side and the contralateral side were evacuated and cleaned by engraving using an endplate curette from the TLIF side. Autografts from the laminectomy site were placed into the anterior part of the evacuated disc space and the TLIF cage (polyetheretherketone). The cage was stabilized in the anterior direction using a hook. Under fluoroscopic guidance, an appropriately tapered rod was applied to the pedicle screws on the TLIF side. After irrigating and assuring hemostasis, a drain was placed, and the incisions were closed.

Statistical analysis

Descriptive statistics were used to define continuous variables (average, standard deviation, minimum, median, maximum). Independent variables with normal distributions were analyzed using the Student’s t-test. Independent variables without a normal distribution were analyzed using the Mann–Whitney U test. In all the analyses, p < 0.05 indicated statistical significance. The statistical analyses were performed using MedCalc Statistical Software (ver. 12.7.7; MedCalc Software bvba, Ostend, Belgium).

## Results

The average age of the 53 patients (20 men, 33 women) was 60.8 years (range: 30–70 years) and the average BMI was 29.9 kg/m^2^ (range: 23.1–39.9 kg/m^2^). Twenty-six patients had BMI < 30 kg/m^2^, while 27 patients had BMI 30–39.9 kg/m^2^. The average BMI of the patients in the BMI < 30 kg/m^2^ group was 26.2 kg/m^2^, while that in the BMI 30–39.9 kg/m^2^ group was 33.6 kg/m^2^ (Table 1).

**Table 1:** Distribution of patients according to body mass index (BMI) and the mean BMI values of the patients.

BMI < 30 kg/m^2^	BMI 30-39.9 kg/m^2^
Number of Patients	26	27
Average BMI	26.2 kg/m^2^	33.6 kg/m^2^

The TLIF level of the BMI 30–39.9 kg/m^2^ group was L4–5 in 17 patients and L5–S1 in 10 patients. The TLIF level of the BMI < 30 kg/m^2^ group was L4–5 in 11 patients and L5–S1 in 15 patients (Table 2).

**Table 2:** Transforaminal lumbar interbody fusion levels in patients with body mass index (BMI) < 30 kg/m2 and BMI 30–39.9 kg/m2. TLIF: Transforaminal lumbar interbody fusion

TLIF level	BMI < 30 kg/m^2^	BMI 30-39.9 kg/m^2^
L4-L5	11	17
L5-S1	15	10

The average volume of blood transfused was 1.9 IU in the BMI < 30 kg/m^2^ group and 1.7 IU in the BMI 30–39.9 kg/m^2^ group. The average Hb/Hct values in the BMI < 30 kg/m^2^ group were 12.9/38.7, while the corresponding values were 12.4/38.03 in the BMI 30–39.9 kg/m^2^ group. The average operative time was 3.6 hours in the BMI < 30 kg/m^2^ group and 3.5 hours in the BMI 30–39.9 kg/m^2^ group. The average blood loss was 488.8 cc in the BMI < 30 kg/m^2^ group and 511.8 cc in the BMI 30–39.9 kg/m^2^ group. There were no statistically significant differences in the volume of blood transfused, preoperative Hb/Hct, operative time, or volume of blood loss between the two groups. The average fluoroscopy time was 0.84 minutes in the BMI < 30 kg/m^2^ group and 1.19 minutes in the BMI 30–39.9 kg/m^2^ group. The average skin incision length was 8.85 and 12.15 cm in the BMI < 30 kg/m^2^ group and BMI 30–39.9 kg/m^2^ group, respectively. These differences in fluoroscopy time and incision length between the two groups were significant (p = 0.01, p < 0.001; Table 3).

**Table 3:** Differences in parameters according to body mass index (BMI) (Mann–Whitney U test, p < 0.05). ​​​​​

	BMI < 30 kg/m^2^	BMI 30-39.9 kg/m^2^	p
	Mean +/- SD	Mean +/- SD	
	Med (range)	Med (range)	
Volume of Blood Transfusion	1.9 +/- 0.9	1.72 +/- 0.8	0.562
	2 (1-3)	2 (1-3)	
Preop Hb	12.9 +/- 1.8	12.4 +/- 1.6	0.247
	12.8 (9.1-15.7)	12.4 (9.5-17.2)	
Preop Hct (%)	38.7 +/- 4.7	38.03 +/- 4.1	0.581
	38.05 (27.8-47.5)	38.9 (28.7-48)	
Operative Time (h)	3.6 +/- 0.8	3.5 +/- 0.9	0.932
	3 (3-6)	3 (3-7)	
Volume of Blood Loss	488.8 +/- 254.8	511.8 +/- 189.05	0.710
	400 (200-1300)	450 (200-850)	
Fluoroscopy Time (min)	0.84 +/- 0.4	1.19 +/- 0.53	0.010
	0.85 (0.3-1.5)	1.1 (0.4-3)	
Skin Incision Length (cm)	8.85 +/- 0.8	12.15 +/- 0.9	<0.001
	9 (8-10)	12 (10-14)	

No complications were observed during the intra-operative period. Seven patients had superficial wound infection. Two of these patients were in the BMI < 30 kg/m^2^ group and five were in the BMI 30–39.9 kg/m^2^ group. Superficial wound debridement was performed in three of five patients and all of these patients were in the BMI 30–39.9 kg/m^2^ group. Five pedicle screws in patients with BMI 30–39.9 kg/m^2^ group were lateral oriented in C-arm fluoroscopy control. The direction of the pedicle screws was revised intra-operatively.

## Discussion

To our knowledge, this is the first study to compare the effects of BMI on intra-operative parameters in obese and non-obese patients. Obesity is a significant factor in the initial clinical and radiological findings associated with lumbar spine disease [9]. Although there have been reports regarding the effects of obesity on surgical outcome [10-14], no study on TLIF procedure has compared the peri-operative data of patients with BMI < 30 kg/m^2^ to peri-operative data of patients with BMI 30–39 kg/m^2^. TLIF surgery has been performed for many years and is a generally accepted procedure [6]. From the surgeon’s viewpoint, surgical exposure is more difficult in obese patients than in non-obese patients owing to the requirement for more retraction and tissue dissection. Consequently, surgical techniques are more challenging, and the rates of intra- and postoperative complications may be higher. Therefore, surgeons must be aware of the increased risk of multiple complications in obese patient population [12,15]. Increased rates of surgical site infections related to increased subcutaneous fatty tissue have been reported by several authors [16-18]. In our study, seven patients had superficial wound infection. Three patients underwent superficial debridement and antibiotic treatment; all of these patients were in the BMI 30–39.9 kg/m^2^ group.

Burks et al. reported an association between an increased incidence of dural failure and obesity in a study involving three different spinal surgical methods, and they found significant differences in the obese group [19]. Difficulties regarding the placement of pedicle screws in obese patients have also been reported in the literature, along with a higher frequency of facet joint damage [20]. Guiding the pedicle screw to the medial area and placing the TLIF cage into disc space securely may be more difficult in obese patients owing to the presence of excess subcutaneous fatty tissue. To prevent lateral wall perforation during pedicle screw placement in obese patients and to achieve accurate and safe TLIF cage placement, we performed further lateral dissections in our obese patients to retract the subcutaneous fatty tissue further laterally. This technique can provide the appropriate medialization of the pedicle screw; furthermore, since the TLIF cage is placed laterally, the hand can be tilted from the lateral to medial side to obtain a convenient and safe interval. Although further lateral dissection was noted in obese patients, the pedicle screw orientation was determined laterally by the C-arm fluoroscopy in three patients with BMI of 30-39.9 kg/m^2^ in our study, and the direction of the pedicle screws was revised intra-operatively. It is assumed that more dissection could cause more bleeding. However, with appropriate surgical techniques and a subperiosteal approach, we did not find any significant increase in bleeding in our patients with BMI 30–39 kg/m^2^. The BMI 30–39 kg/m^2^ group had approximately 50 cc greater blood loss than the BMI < 30 kg/m^2^ group; however, this difference was not statistically significant, which may have been associated with wider skin incisions and further lateral dissection. On the basis of the results in the literature, the average volume of bleeding during the single-level TLIF procedure is 400–600 mL [11,21,22]. The amount of bleeding in our study was consistent with that reported in the literature. We think that the difference in skin incision length between the two groups is due to the lateral dissection technique applied in obese patients and that subcutaneous adipose tissue needs further retraction to provide better surgical visualization. Marquez et al. reported that the operative time was 11 minutes longer in obese versus non-obese patients [12]. They set the BMI limit to 25 kg/m^2^ and the surgical levels of obese patients were longer than those in other patients. Andreshak et al. found no differences in operative times between obese and non-obese individuals [23]. Another study showed that the duration of surgery was longer in morbidly obese patients than in obese patients [24]. The patients in our study had single-level illness and all were treated by the same surgeon. In contrast to the literature and our expectations, the surgical procedure time was 6 minutes longer in the BMI < 30 kg/m^2^ group. This may have been because of the greater number of cases involving the L5–S1 level than the L4–L5 level in the BMI < 30 kg/m^2^ group. Although there have been no previous studies comparing TLIF procedures at the L5–S1 and the L4–L5 levels, in our experience, downward angulation of the L5–S1 disc in the lateral plane may complicate endplate preparation. This could explain the difference in surgical time between the two groups. In this study, the statistically significant difference between the two groups was found in the duration of fluoroscopy, which was 35 s longer in the BMI 30–39.9 kg/m^2^ group than in the BMI <30 kg/m^2^ group. In the literature, it has been mentioned that annual dose limits can be exceeded if fluoroscopic-guided procedures such as minimally invasive TLIF interventions are performed in large numbers [25,26]. As the health of the surgeon and his team as well as patients is important, it should be kept in mind that more radiation exposure occurs during the TLIF procedure in obese patients and that necessary preventions should be taken.

The effects of obesity on the clinical results after spinal surgery have been evaluated using the visual analog scale (VAS) score and the Oswestry Disability Index (ODI) in other studies [10,27]. One of the limitations of our study is the absence of VAS and ODI data. However, the main outcome of our study was not the clinical outcome of the TLIF procedure in obese patients but to evaluate the intra-operative parameters and difficulties encountered during TLIF. Another limitation of our study is the lack of further classification of the patients with BMI < 30 kg/m^2^. These patients could have been further divided into subgroups according to whether their BMI was lower or higher than 25 kg/m^2^, but the number of patients included in our study population was not sufficient for such an analysis. Despite these limitations, the positive aspects of our study are that all patients were treated by a single surgeon and the same surgical team, and the same surgical procedure was applied in all cases.

## Conclusions

An experienced surgical team, taking appropriate precautions with respect to certain surgical details, can safely apply the TLIF procedure in patients with obesity. However, obesity should be taken into consideration by surgeons before TLIF procedure and some modifications in the surgical technique, including far lateral dissection of soft tissue for pedicle screw medialization and secure TLIF cage placement, may be required during the procedure. Furthermore, it should be kept in mind that both the surgical team and the patient may be exposed to radiation for a longer period when performing TLIF in obese patients than in non-obese patients.
